# Determinants Outside the DevR C-Terminal Domain Are Essential for Cooperativity and Robust Activation of Dormancy Genes in *Mycobacterium tuberculosis*

**DOI:** 10.1371/journal.pone.0016500

**Published:** 2011-01-27

**Authors:** Uma Shankar Gautam, Santosh Chauhan, Jaya Sivaswami Tyagi

**Affiliations:** Department of Biotechnology, All India Institute of Medical Sciences, New Delhi, Indian; Charité-University Medicine Berlin, Germany

## Abstract

**Background:**

DevR (also called as DosR) is a two-domain response regulator of the NarL subfamily that controls dormancy adaptation of *Mycobacterium tuberculosis* (*M. tb*). In response to inducing signals such as hypoxia and ascorbic acid, the N-terminal receiver domain of DevR (DevR_N_) is phosphorylated at Asp54. This results in DevR binding to DNA *via* its C-terminal domain (DevR_C_) and subsequent induction of the DevR regulon. The mechanism of phosphorylation-mediated activation is not known. The present study was designed to understand the role of the N- and C-terminal domains of DevR in DevR regulon genes activation.

**Methodology/Principal Findings:**

Towards deciphering the activation mechanism of DevR, we compared the DNA binding properties of DevR_C_ and DevR and correlated the findings with their ability to activate gene expression. We show that isolated DevR_C_ can interact with DNA, but only with the high affinity site of a representative target promoter. Therefore, one role of DevR_N_ is to mask the intrinsic DNA binding function of DevR_C_. However, unlike phosphorylated DevR, isolated DevR_C_ does not interact with the adjacent low affinity binding site suggesting that a second role of DevR_N_ is in cooperative binding to the secondary site. Transcriptional analysis shows that consistent with unmasking of its DNA binding property, DevR_C_ supports the aerobic induction, albeit feebly, of DevR regulon genes but is unable to sustain gene activation during hypoxia.

**Conclusions/Significance:**

DevR is a unique response regulator that employs a dual activation mechanism including relief of inhibition and cooperative interaction with binding sites. Importantly, both these functions reside outside the C-terminal domain. DevR_N_ is also essential for stabilizing DevR and sustaining autoregulation under hypoxia. Hence, both domains of DevR are required for robust transcription activation.

## Introduction

Bacterial persistence is a hallmark of tuberculosis (TB). Following a TB infection, the individual usually mounts an effective immune response that leads to a cessation of disease progression due to the formation of granulomas around infective foci. Clinical studies suggest that the bacilli within these granulomas remain dormant in untreated individuals, causing latent infection that can last a lifetime [1], [2]. Oxygen limitation during granuloma development has been proposed to be one of the main signals that alter the metabolic status of bacteria to a state of dormancy [3]. Two-component systems are majorly involved in sensing and responding to changing environments in bacteria [4]. Numerous studies have demonstrated the relevance of the DevR-DevS two-component system in virulence and adaptation of *Mycobacterium tuberculosis* (*M. tb*) to putative granuloma signals including hypoxia, nitric oxide, carbon monoxide and ascorbic acid [5]–[13]. It mediates the induction of ∼48 genes referred to as the DevR regulon [8] and this genetic response is essential for bacterial adaptation and persistence under hypoxia [14], [15].

DevR (*Rv3133c*, also called DosR) is one of the best characterized transcriptional regulators of *M. tb*. It is a typical two-domain response regulator of the NarL subfamily [5] and its N-terminal domain that contains a phosphorylation site, Asp54, is connected to the C-terminal DNA binding domain (DevR_C_) by a linker sequence [16]–[19]. The target genes of the DevR regulon were predicted to contain one, two or more putative DevR binding sites (Dev boxes) in their upstream regions [8]. We have shown the importance of cooperative binding of DevR to two or more sites for the full induction of some of these genes. Close packing of the binding sites and an overlap of the Transcription start point (TSP)-proximal binding site with the -35 promoter element were common features of the target promoters that were analyzed [20]–[22]. While DevR_C_ interaction with DNA oligonucleotides containing two consensus binding sequences was shown by crystal structure analysis [18], phosphorylation of intact DevR at Asp54 was found to be essential for interaction with DNA [20]. The importance of phosphorylation was supported by visualizing extensive interactions between the N- and C-terminal domains in the DevR structure that mask the DNA binding domain. A helix rearrangement mechanism was proposed to alleviate this inhibition [19].

The present study was designed to understand the role of the N- and C-terminal domains in activation of the DevR regulon genes. We compared the DNA binding properties of DevR_C_ and DevR and correlated the findings with their ability to activate gene expression. We show that DevR_C_ activates albeit weakly, the aerobic expression of the DevR regulon. The inability of DevR_C_ to support full induction is attributed, at least in part, to a failure to cooperatively recruit DevR to adjacently-placed secondary binding sites. We also show that *devR_C_* transcript and DevR_C_ protein levels are not maintained during hypoxia. The present study reveals the multifunctional role of the DevR_N_ domain. In addition to receiving the phosphosignal at Asp54 from DevS and DosT kinases [16], [17], [23], DevR_N_ suppresses the DNA binding and transcription-activating ability of unphosphorylated DevR under aerobic conditions, sponsors cooperative binding of DevR with secondary binding sites, and it is required for sustaining DevR stability and autoregulation during hypoxia. Thus DevR action is mediated by both its N-terminal and C-terminal domains.

## Materials and Methods

### Plasmids, bacterial strains, and culture conditions

All plasmids and bacterial strains used in this study are described in Tables 1 and 2, respectively. *M. tb* strains were cultured at 37°C in Dubos medium containing 0.05% Tween-80 plus 0.5% albumin, 0.75% dextrose and 0.085% NaCl (DTA medium). *Escherichia coli* (*E. coli*) strains and culture conditions were as described earlier [20]. Antibiotics were used at the following concentrations: hygromycin at 50 µg/ml for *M. tb* and 200 µg/ml for *E. coli*, kanamycin at 20 µg/ml for *M. tb* and 50 µg/ml for *E. coli*.

**Table 1:** Plasmids used in this study.

Plasmid	Relevant features^a^	Source/Reference
pUS-DevR_C_	pET28a overexpressing DevR C-terminal domain cloned in NdeI site	This study
pAV-DevR	pET28a overexpressing full length wild type DevR cloned in NdeI site	[12]
pJFR19	*E. coli*-Mycobacterium integrating shuttle plasmid with 3-kbacetamidase promoter, Hygr	[43]
pFPV27	*E. coli*-Mycobacterium shuttle plasmid with promoter less *gfp*, Kanr	[44]
pET28a	*E. coli* expression vector (with N-terminal His_6_ tag), Kanr	Novagen
pMG86	pJFR19 containing *devR*-*devS* expressed from acetamidase promoter, Hygr	[45]
pTGS	pFPV27 containing *tgs1* promoter (-143 to +45), Kanr	[22]
p3131	pFPV27 containing *Rv3131* promoter (-150 to +48), Kanr	[22]
pSD P_Operon_ *devR*	pJFR19 containing *devR* (cloned between NdeI and XbaI sites), full-length DevR is expressed from *Rv3134c-devRS* operon promoter (-608 to +998, ref. 20) cloned in NdeI and BstBI sites	S.D.Majumdar and J.S.Tyagi, 2010, unpublished
pUS P_Operon_ *devR_C_*	pJFR19 containing *devR_C_* (cloned between NdeI and XbaI sites), DevR_C_ is expressed from *Rv3134c-devRS* operon promoter cloned in NdeI and BstBI sites	This study
pUS P_Acet_ *devR_C_*	pJFR19 containing *devR_C_* (cloned between NdeI and XbaI sites), DevR_C_ expressed from constitutive acetamidase promoter cloned in NdeI and BstBI sites	This study
pUS P_hsp60_ *devR_C_*	pJFR19 containing *devR_C_* (cloned between NdeI and XbaI sites), DevR_C_ expressed from constitutive *hsp60* promoter cloned in NdeI and BstBI sites	This study

a The coordinates of the promoters (in parentheses) are with reference to the transcription start point (TSP) of *tgs1*;

Hyg^r^, hygromycin resistance;

Kan^r^, kanamycin resistance.

**Table 2:** Strains used in this study.

*M. tb* strain	Relevant features	Source/Reference
H37Rv	WT laboratory strain of *M. tuberculosis* (*M. tb*)	Laboratory collection
Δ*devR*	447-bp BalI deletion in *M. tb* H37Rv *devR* gene (deletes DevR amino acid residues from position 40 to191)	[46]
Comp13	Δ*devR* complemented with plasmid pSD P_Operon_ *devR*, expresses full-length DevR protein	S.D. Majumdar,Ph.D. ThesisSubmitted, 2010
Comp5	Δ*devR* complemented with plasmid pUS P_Operon_ *devR_C_*, expresses DevR_C_ protein	This study
Comp6	Δ*devR* complemented with plasmid pUS P_Acet_ *devR_C_*, expresses DevR_C_ protein	This study
Comp7	Δ*devR* complemented with plasmid pUS P_hsp60_ *devR_C_*, expresses DevR_C_ protein	This study

### Construction of DevR_C_ over-expressing plasmid and purification of DevR_C_

The *devR* C-terminal domain coding sequence (141-217 amino acids of DevR) was amplified from *M. tb* H37Rv DNA by PCR (Table 3), and cloned into pET28a to generate pUS-DevR_C_ which expresses the C-terminal domain. N-terminal His_6_-tagged DevR_C_ and full-length DevR proteins (referred to as DevR_C_ and DevR, respectively) were overexpressed in *E. coli* C43 (DE3) from pUS-DevR_C_ and pAV-DevR, respectively using standard procedures. The recombinant proteins were purified by standard techniques and used in EMSA and DNase I footprinting experiments.

**Table 3:** Primers used in this study.

Primer	Sequence (5′→ 3′)	Application
devR_C_ NdeI F	CGGACCCATATGCAGGACCCGCTATCAGGC	Cloning of *devR_C_* in pJFR19
devR_C_ XbaI R	CCGCTCTAGACCTGTTGTCATGGTCCATCACCGGGTG	
hsp60 BstBI F	CCGTTCGAAGGTGACCACAACGACGCGCCCGC	Cloning of *hsp60* promoter in pJFR19
hsp60 NdeI R	CCGCATATGTGCGAAGTGATTCCTCCGGATCG	
devR_C_ NdeI F	CGGACCCATATGCAGGACCCGCTATCAGGC	Cloning of *devR_C_* in pET28a
devR_C_ NdeI R	CCGCATATGCTATCATGGTCCATCACCGGGTGG	
RT16S F	ATGACGGCCTTCGGGTTGTAA	Real Time RT PCR (ref. 13)
RT16S R	CGGCTGCTGGCACGTAGTTG	Real Time RT PCR (ref. 13)
RT3134c F	CTGGCTGGGTCGGCCTTAGC	Real Time RT PCR (ref. 13)
RT3134c R	TGACCTGGGAGGTTGTCG	Real Time RT PCR (ref. 13)
RTdevR_C_ F5	CGAGGATCCCTGTTGTCATGGTCCAT	Real Time RT PCR
RTdevR R	CGCGGCTTGCGTCCGACGTTC	Real Time RT PCR
RT devS F	TACTGACCGACCGGGATCGT	Real Time RT PCR (ref. 13)
RTdevS R	AGAGCCGCTGGATGACATGG	Real Time RT PCR (ref. 13)
RT1738 F	CGACGAACACGAAGGATTGA	Real Time RT PCR
RT1738 R	ACACCCACCAATTCCTTTTCC	Real Time RT PCR
RT2031c F	CGCACCGAGCAGAAGGA	Real Time RT PCR
RT2031c R	ACCGTGCGAACGAAGGAA	Real Time RT PCR
RTtgs1 F	CAGTGATTTGCGTCGCTACAG	Real Time RT PCR
RTtgs1 R	ACATCATTGATGGTGACGTCG	Real Time RT PCR
RT3131 F	CGATCAGGCCGATGTCGCCTT	Real Time RT PCR
RT3131 R	TCACCTCCTGGCACCGGCC	Real Time RT PCR
LH1	CGAGTCGACAGAGCACGAAGGCTCGCCAGCGGAGG ACCTTTGGCCCTGCGTCGACCGA	Gel shift assays (P+S box) (ref. 22)
LH2	TCGGTCGACGCAGGGCCAAAGGTCCTCCGCTGGCGA GCCTTCGTGCTCTGGTCGACTCG	Gel shift assays (P+S box) (ref. 22)
3130F	TGGCTGCCGGGCCTTTCCCAT	DNase I footprinting (ref. 22)
3131R	CATGGTCAGCGCCTTCCCCGG	DNase I footprinting (ref. 22)

NdeI, XbaI, and BstBI restriction enzyme sites are underlined.

### Construction of *M. tb* strains expressing DevR_C_

The gene sequences encoding the DevR C-terminal domain were amplified from H37Rv DNA by PCR. The amplified DNA was cloned into the integrative plasmid pJFR19 to generate pUS P_Acet_ *devR_C_*. For DevR_C_ expression from the native *Rv3134c-devRS* operon promoter, the operon promoter was excised from plasmid pSD P_Operon_ *devR* and cloned upstream of the DevR_C_-coding sequence to generate pUS P_Operon_ *devR_C_*. For expression from the *hsp60* promoter, the operon promoter (in pUS P_Operon_ *devR_C_*) was replaced with the *hsp60* promoter to generate plasmid pUS P_hsp60_ *devR_C_*, These integrating plasmids, namely, pUS P_Operon_ *devRc*, pUS P_Acet_ *devR_C_*, and pUS P_hsp60_ *devRc* were individually electroporated into *M. tb* Δ*devR* bacteria to generate Comp5, Comp6 and Comp7 strains, respectively (Table 2).

### Western blotting

Frozen stocks of *M. tb* strains were revived in DTA medium, subcultured thrice and grown with vigorous shaking (120 ml in a 500-ml flask) in a shaker incubator at 220 rpm till ∼*A*_595_ 0.2-0.3, and subsequently processed for immunoblotting and RNA analysis (below). Briefly, a 20 ml aliquot was chilled on ice (‘aerobic’), centrifuged immediately at 5,000 g for 10 min at 4°C and the pellet was stored at −20°C. Sixty ml of the culture was distributed (10 ml aliquots in 50 ml tubes that were tightly closed) and kept standing for 1, 3 and 5 days (‘hypoxic’). The cells were harvested from dedicated culture tubes after appropriate incubation and whole cell lysates were prepared as described [24]. SigA protein was used as internal control. HspX and SigA proteins were detected in the lysates (containing ∼15 µg protein) by western blotting using polyclonal anti-HspX and anti-SigA antibodies as described earlier [25].

### RNA isolation

The remaining culture (40 ml from above) was harvested and RNA was isolated. Briefly, a 20 ml aliquot was chilled on ice and centrifuged immediately as described above (‘aerobic’) and the remaining culture was kept standing for 5 days (‘hypoxic’). The harvested cell pellets were each resuspended in 1 ml of TRI reagent (Molecular Research Center, USA), and lysed in a mini bead beater using 0.1 mm zirconium/silica beads (Biospec, USA). RNA was purified as described earlier [20].

### Reverse transcription (RT) and Real Time PCR

Two hundred nanograms of DNA-free RNA was reverse transcribed into cDNA using 50 U of Multi Scribe reverse transcriptase and random hexamer primers as per manufacturer's instructions (Applied Biosystems, USA). The cDNA was subjected to real time PCR using gene specific primers (Table 3) and *Power* SYBR Green PCR Master Mix in a MyiQ thermal cycler (Bio-Rad, USA). The primers were designed using the Primer3 program (http://workbench.sdsc.edu) and gene sequence data obtained from TubercuList (http://genolist.pasteur.fr/TubercuList). Reaction conditions were 94°C (10 min) followed by 40 cycles of 94°C (30 s), 56–65°C (45 s) and 72°C (30 s). A RT-negative (without Reverse Transcriptase) reaction was used to account for residual DNA if any and transcript numbers were normalized to that of 16S rRNA. The normalized copy number values were then used to determine the relative quantities (RQ) of individual gene transcripts. Three independent cultures were each analyzed in duplicate and the results are expressed as Mean ± SD.

### EMSA and DNase I footprinting

The binding patterns of full-length DevR and DevR_C_ proteins were compared in EMSA and DNase I footprinting experiments. EMSA assays were performed with purified DevR or DevR_C_ protein and DNA fragments containing double-stranded oligonucleotides corresponding to the P+S binding sites located in the *tgs1-Rv3131* intergenic promoter region. When used, full-length DevR was phosphorylated by incubating it with 50 mM acetyl phosphate for 20 min at 25°C in 40 mM Tris-Cl (pH 8.0) and 5 mM MgCl_2_. EMSA and DNase I footprinting analysis were carried out as described previously [22]. The sequences of the primers used in EMSA and DNase I footprinting are shown in Table 3.

### GFP reporter assay

Aerobic GFP reporter assays were conducted in DTA medium as described previously [20]. The promoter activity is expressed in Relative Fluorescence Units (RFU)/OD_595_ of GFP as Mean values of RFU/OD ± standard deviation of three independent experiments, each in triplicate.

## Results

Towards determining the role of the C-terminal domain of DevR in transcriptional regulation, we analyzed the in vitro binding property of its isolated C-terminal domain, DevR_C_. We also compared DevR_C_ and full-length DevR proteins with respect to their ability to activate the transcription of target genes.

### Isolated DevR_C_ domain interacts with DNA but is deficient in cooperative interactions

**Figure 1:**
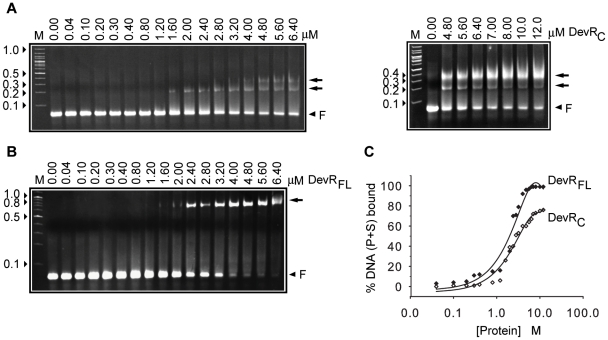
EMSA analysis. Interaction of DevR_C_ (**A**) and full-length DevR (**B**), with *tgs1-Rv3131* promoter DNA. Double-stranded oligonucleotides having P+S box sequences belonging to the *tgs1-Rv3131* divergent promoters were incubated with increasing concentrations of DevR_C_ or DevR. Arrow, DNA-protein complex; F, free oligonucleotides, arrowheads indicate molecular weight markers in kilobase pairs (lane M), (**C**) Fraction of bound DNA (from Fig. 1A, B) plotted against protein concentration.

**Figure 2:**
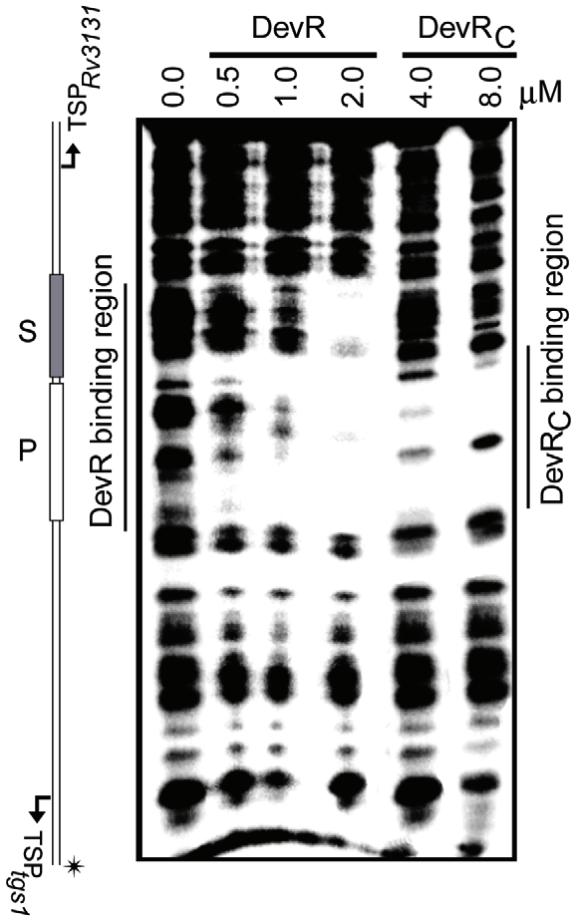
DevR_C_ is defective in cooperative binding to DNA. DNase I footprinting of DevR_C_ or DevR on *tgs1-Rv3131* intergenic DNA containing P and S binding sites. Bent arrows indicate the positions of the TSPs. ^32^P radiolabeled DNA strand is indicated by an asterisk.

The *tgs1-Rv3131* intergenic promoter region was used to assess DevR_C_ interaction with DNA because these divergent promoters are regulated by DevR interaction with two binding sites, P and S [22]. At first, EMSA assays were carried out using double-stranded oligonucleotides containing P and S binding sites (called as P+S). The interaction between DevR_C_ and P+S DNA generated two progressive DevR_C_-DNA complexes; first, a faster moving species (alongside ∼200 bp DNA marker), was observed and subsequently a slower migrating complex (alongside <400 bp DNA marker), also appeared at higher protein concentrations (Fig. 1A). This was significantly different from the interaction of full-length DevR which produced a single DNA-protein complex of low mobility that migrated alongside ∼700 bp DNA marker without an intermediate species, even at low protein concentrations, suggesting the interaction to be strongly cooperative (Fig. 1B). Another notable difference was that only partial saturation of DNA was observed even at very high concentration of DevR_C_ (Fig. 1A, 1C). DNase I footprinting analysis of DevR_C_ with the *tgs1-Rv3131* intergenic region revealed that it binds to the primary site, but fails to cooperatively bind to the adjacent site, unlike full-length DevR which protected both the sites (Fig. 2). The underlying reason for obtaining two bound complexes with DevR_C_ in EMSA is not well understood. Because the S site is not bound to DevR_C_, the appearance of the slower migrating species at higher protein concentration (>3 µM) is likely to be a result of interactions involving P-site bound DevR_C_ species.

### DevR_C_ feebly activates DevR regulon gene expression under aerobic conditions

**Figure 3:**
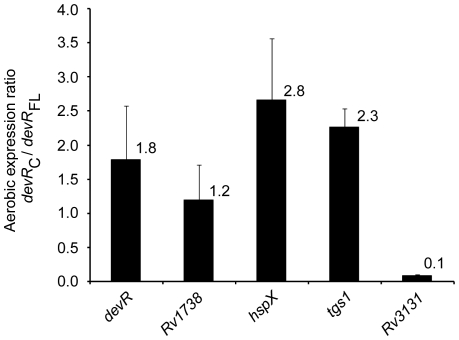
Aerobic expression of selected DevR target genes. The relative aerobic transcript levels of selected genes were estimated by real time RT-PCR analysis in DevR_C_-expressing Comp 5 bacteria and expressed in relation to that in aerobic Comp13 cultures (expressing full-length DevR).

To address whether the DevR N-terminal domain has a regulatory role in suppressing DevR regulon gene expression under aerobic conditions, we asked whether DevR_C_ could activate transcription in the absence of the inducing signal (i.e. under aerobic conditions). For this, we compared the relative quantities of *tgs1*, *Rv3131* and selected DevR regulon transcripts in aerobic *M. tb* cultures of similar genetic background that produce DevR_C_ (Comp5 strain) or full-length protein (Comp13 strain) from an identical chromosomal location. An ∼2-fold higher level of aerobic *devR* transcripts was estimated in Comp5 vs. Comp13 bacteria (Fig. 3), demonstrating that DevR_C_ autoregulates transcription in aerobic cultures. This is noteworthy because in WT DevR-expressing cultures, autoregulation is dependent on DevR phosphorylation which occurs under hypoxic and not under aerobic conditions [20]. The expression of a target gene, *tgs1*, was also elevated > 2 fold in DevR_C_-expressing aerobic cultures (Fig. 3) and this aerobic overexpression was confirmed by GFP reporter assay using pTGS (mean aerobic GFP fluorescence ∼450 RFU/OD vs. ∼42 RFU/OD in the presence of WT DevR). The expression of various other DevR regulon genes was also induced, albeit modestly, in aerobic Comp5 bacteria (upto ∼3-fold, Fig. 3).

### DevR_C_ supports the aerobic expression of HspX

**Figure 4:**
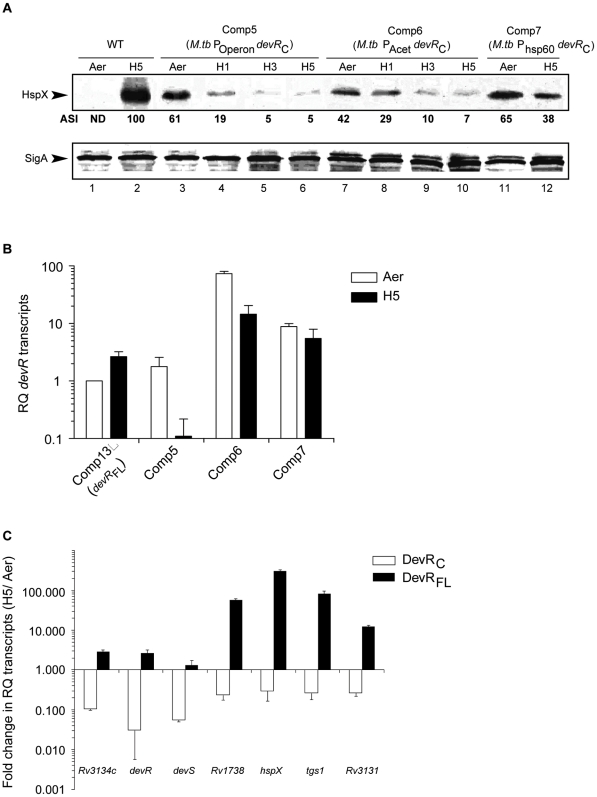
DevR regulon expression in DevR_C_-expressing cultures declines during hypoxia. (**A**) *M. tb* lysates (15 µg protein) were immunoblotted using rabbit anti-HspX or anti-SigA polyclonal sera and the blots were analyzed using Quantity One software (Bio-Rad, USA). The normalised intensities of the HspX-derived signals (with respect to those of SigA) are denoted as Arbitrary Signal Intensities (ASI) with respect to those obtained in 5 days hypoxic WT cultures (H5). ‘Aer’, aerobic; H1 H3 and H5 refer to 1, 3 and 5 days hypoxic cultures, respectively; ND, not detected. (**B**) Relative Quantity (RQ) of *devR_C_* transcripts in different Comp strains determined by real time RT-PCR analysis. (**C**) Real time RT-PCR analysis of DevR regulon transcripts. Fold change in the relative quantity of transcripts under ‘hypoxic’ vs. ‘aerobic’ conditions (**fold decrease** in Comp5 and **fold increase** in Comp13) is shown.

The ability of DevR_C_ to mediate gene induction was confirmed at the protein level. HspX protein was detected in aerobic DevR_C_-expressing *M. tb* strains (Comp5), but not in aerobic cultures expressing full-length protein. Because HspX expression is DevR dependent, its expression implies the presence of an adequate amount of DevR_C_ in aerobic Comp cultures (Fig. 4A). However, despite analyzing a large quantity of protein by immunoblotting (∼80 µg), DevR_C_ was undetectable in Comp bacterial lysates (see Discussion). An artefactual increase in HspX expression during centrifugation of DevR_C_-expressing *M. tb* was ruled out by the absence of HspX expression in aerobic WT cultures that were processed in parallel. Moreover, activation by phosphorylation (ie. during hypoxia/centrifugation) is not relevant for DevR_C_ because it lacks the phosphorylatable N-terminal domain.

### DevR regulon induction is not sustained by DevR_C_ during hypoxia

Since DevR is physiologically relevant for regulon induction during hypoxia, the ability of DevR_C_ to support hypoxic expression was examined next. The results of qRT-PCR and western blot analysis demonstrate that contrary to wild type DevR-expressing bacteria, gene induction is not sustained during hypoxia in DevR_C_-expressing Comp 5 bacteria (Fig. 4). An approximately 3- to 18-fold reduction in *devR* transcripts and regulon transcripts was observed in hypoxic Comp5 cultures in striking contrast to an ∼2- to 300-fold increase in Comp13 bacteria (expressing full-length DevR) under identical conditions (Fig. 4C). These results establish that DevR_C_-expressing bacteria have an autoregulation defect and an associated defect in regulon induction under hypoxia. The induction defect was also noted at the level of protein expression; HspX protein levels progressively decreased by >10-fold in Comp5 bacteria over 5 days in contrast to the sustained hypoxic induction noted in WT *M. tb* cultures (Fig. 4A, lanes 5 and 6). The decrease in HspX protein levels in Comp5 bacteria paralleled the decline in *hspX* transcripts on day 5 (Fig. 4C). As expected, SigA was constitutively expressed in all the strains under aerobic and hypoxic conditions.

Possible reasons for the decline in DevR regulon expression in hypoxic Comp5 cultures are that *devR_C_* transcripts are unstable or poorly expressed from the native promoter and therefore unable to maintain DevR_C_ levels at a level adequate for autoregulation and target genes induction during hypoxia. To address these questions, two additional *M. tb* strains, Comp6 and Comp7, were constructed wherein DevR_C_ is expressed from the constitutive acetamidase and *hsp60* promoters, respectively, each with its own translational signals. Note that Comp6 and Comp7 are identical to Comp5 except for the promoter that is used to transcribe *devR_C_*. Transcription from the acetamidase and *hsp60* promoters (in Comp6 and Comp7 bacteria) did enhance *devR_C_* transcript levels; the relative quantity (RQ) of *devR_C_* transcripts increased to ∼15 and ∼5, respectively, vs. <0.2 in Comp5 cultures (Fig. 4B). However, in spite of an increase in *devR_C_* transcripts during hypoxia, HspX levels were not sustained, particularly in Comp6 cultures (Fig. 4A, lanes 9 and 10), and the expression of other genes of the regulon also declined in these strains during hypoxia (data not shown). Therefore, we infer that DevR_C_ is not stable at the protein level in the absence of DevR_N_ in *M. tb* cultures. These results are in contrast to WT bacteria wherein DevR regulon products are induced and maintained during the 5-day hypoxia period. We conclude that in addition to the cooperativity defect, likely reasons for the failure of the hypoxic response are the selective instability of truncated DevR_C_ protein and/or inability of DevR_C_ to support its own transcription owing to missing of crucial interactions with the transcription machinery in Comp bacteria.

## Discussion

Recently we showed by analyzing some target genes of the DevR regulon that robust induction depends on the binding of native phosphorylated DevR protein to two or more binding sites located in target promoters [20]–[22]. A DevR_C_-DNA complex was visualized by others from crystal structure analysis [18], and therefore we hypothesized that perhaps DevR_C_ could support robust aerobic expression of DevR regulon genes. To address this possibility, we characterised the isolated C-terminal domain of DevR with respect to its DNA binding properties in vitro and its role in transcriptional activation in vivo. In the present study, expression analysis suggests that DevR_C_ does indeed support aerobic gene expression, but only at a modest level. An analysis of the arrangement of Dev boxes at target promoters and the pattern of their occupancy provides insights into the underlying defect. We show that DevR_C_ does bind to DNA but it is not recruited to the adjacent binding site at a target promoter unlike intact DevR protein. This difference in binding property is crucial because we know that complete occupancy of the binding sites is functionally important for full induction [22]. For example, DevR_C_ does not bind to the S box in the *tgs1-Rv3131* intergenic region and this defect is associated with the lack of *Rv3131* aerobic expression. Taking together the results of previous and present findings, we attribute the poor aerobic induction of target genes, in fair measure, to the failure of DevR_C_ to mediate cooperative interactions. The target promoters are characterized by an overlap of the TSP-proximal binding site with the -35 promoter element [20]–[22]. Therefore, another possible contributory factor is that interactions between DevR_N_ and RNA polymerase are necessary for transcriptional activation and these are missing in DevR_C_-expressing bacteria. A consideration of all the results supports masking by DevR_N_ of the intrinsic DNA binding activity of DevR_C_ in the intact protein as a regulatory mechanism to prevent the aerobic induction of regulon genes.

We also compared the mechanism of DevR activation with that proposed for other response regulators, including those belonging to the NarL family. Many of the response regulators are placed in one of two classes with respect to the consequences of phosphorylation and mechanism of activation. In the first class, phosphorylation of the N-terminal domain activates the DNA binding activity of the protein by triggering its oligomerisation as in OmpR, ArcA and NtrC [26]–[30]. In the second class, the regulatory domain is believed to act negatively on the DNA binding function and phosphorylation is thought to relieve this inhibition by triggering a conformational change and/or inducing dimerization or oligomerization as in FixJ, PhoB, StyR, NarL and Spo0A [31]–[35]. Indeed, the isolated C-terminal domains of several response regulators, such as FixJ, PhoB, SsrB, Spo0A, and RhaS bind to DNA and activate transcription [31], [32], [36]–[40]. Since intact DevR binds to DNA only upon phosphorylation [20], and isolated DevR_C_ exhibits DNA binding ability (this study), DevR resembles response regulators of the second class and uses domain separation as a key mechanism of activation. Our findings are substantiated by the proposal of domain rearrangement that was made from structural analysis [19]. However, relief of inhibition is not the only mechanism of activation in some response regulators. The isolated C-terminal domain of NarL, a close homologue of DevR, binds to DNA but does not activate transcription [35], implying a regulatory role for its N-terminal domain. NtrC from *Salmonella typhimurium* resembles DevR_C_ in that its C-terminal domain is defective in cooperative interaction and its N-terminal domain is required for this function [29]. However, NtrC differs from DevR in that it binds to DNA as an unphosphorylated protein but its binding efficiency is enhanced by phosphorylation [29]. These comparisons highlight the rich diversity in the activation mechanisms employed by various response regulators, including those belonging to the same family. DevR is a unique example of a regulator that exploits an activation mechanism involving both relief of inhibition and cooperative binding to control gene induction. Importantly, both these functions reside in the N-terminal domain and/or linker region of DevR. As we have not examined it, we cannot rule out the effect of phosphorylation on the oligomerization status of DevR. Additionally, since DevR appears to interact with the transcriptional machinery to activate transcription, the role of the individual domains in these interactions remains to be elucidated.

The sequential binding of DevR to high affinity and low affinity sites may constitute a safety mechanism to tightly regulate induction and prevent regulon activation in the absence of the inducing signals. Since DevR plays a key role in *M. tb* dormancy it is considered to be a novel target for the development of drugs effective against dormant organisms [8], [41]. In principle, DevR-mediated signalling can be intercepted at any of the steps in the signalling cascade, including, signal sensing, DevS/DosT sensor kinase activation, transfer of the phosphosignal to DevR and binding of DevR to target DNA [42]. We recently provided a proof-of-concept for interfering with *M. tb* dormancy by inhibiting DevR activity through a small molecule [15]. Because the present study shows that cooperative binding is crucial for gene activation, hence blocking of cooperativity offers an additional step at which DevR can be effectively intercepted.

In conclusion, the major findings of this study are (i) the intrinsic DNA binding activity of DevR_C_ and aerobic expression of the DevR regulon is masked by DevR_N_, (ii) DevR_C_ fails to interact cooperatively with the binding sites at target promoters, and (iii) DevR_C_ activates transcription of the DevR regulon genes under aerobic conditions, but only weakly, and the induction is not sustained during hypoxia. The binding property of DevR_C_ is in striking contrast to intact phosphorylated DevR, which binds to two or more upstream binding sites and in a highly cooperative manner. From these findings we conclude that the determinant(s) of cooperativity are located outside of the C-terminal domain. These determinants are likely to fulfill a very important function in a genomic context wherein DevR binding sites may vary widely in their strengths; and cooperativity would play a key role in recruiting DevR to all the binding sites at target promoters. In addition to cooperativity, these determinants also provide other vital and essential functions that include autoregulation during hypoxia as well as imparting stability to DevR protein and providing surfaces for interacting with the transcriptional machinery. Thus, the activity and function of DevR is determined by both its C-terminal and N-terminal domains.
